# Acetabular Reconstruction Using Multiple Porous Tantalum Augments: Three-Quarter Football Augment

**DOI:** 10.1155/2022/7954052

**Published:** 2022-05-21

**Authors:** C. H. Ansorge, M. Ohlmeier, T. M. Ballhause, T. Gehrke, M. Citak, M. Lee

**Affiliations:** ^1^Department of Joint Surgery, Helios ENDO-Klinik Hamburg, Holstenstrasse 2, 22767 Hamburg, Germany; ^2^Department of Orthopaedics, Nepean Hospital, Penrith, New South Wales, Australia

## Abstract

Reconstruction of a large acetabular bone defect is a complex problem in revision hip arthroplasty. The authors report a novel method of reconstructing an uncontained acetabular defect (Paprosky type IIIb) using multiple tantalum augments. A 73-year-old female patient presented to our institution with a chronically dislocated primary left total hip arthroplasty with radiographs demonstrating migration of acetabular component and formation of pseudoarthrosis within the left ilium. Extensive arthrolysis and anatomic reconstruction of the acetabular bone defect were performed using the novel method of multiple tantalum augments. Postoperatively, recovery was initially complicated by multiple dislocations requiring an exchange to an elevated liner, however subsequently achieved good function.

## 1. Introduction

Anatomic reconstruction of large acetabular defects is a difficult problem in revision arthroplasty, and various methods are published in the literature to address these defects [1]. Principles in the management of acetabular defects include osseous reconstruction using autologous bone graft or allograft [2–6], the use of metallic bone graft substitute [7–16], or the use of bone cement [17, 18]. The surgical algorithm is dependent on the patient's age, size, and type of defect [19]. We would like to describe a novel method of acetabular reconstruction using multiple tantalum augments and bone cement for a patient with a chronically dislocated primary total hip arthroplasty and pseudoarthrosis.

## 2. Case Report

### 2.1. Patient History

**Figure 1:**
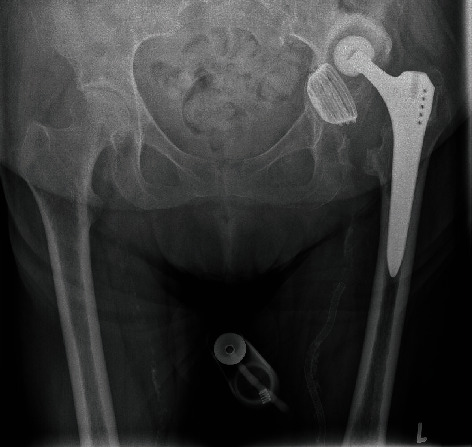
The pelvic radiographs demonstrate dislocation of left total hip replacement, protrusio acetabuli, formation of pseudoarthrosis, and gross loosening of the screw cup. No signs of loosening or fracture of the Zweymüller type femoral stem. Incidental femoral stent on the left.

A 73-year-old female patient presented to our institution with a two-year history of inability to walk on the left hip. The initial primary total hip was performed for osteoarthritis at another institution 10 years ago, after which she had a good function for multiple years. The last radiograph of the left hip was done two years prior, where it was incidentally imaged during a left femoral angiogram. This demonstrated left hip dislocation, migration of acetabular component, and formation of pseudoarthrosis within the left ilium (Figure 1). The subsequent surgical management was delayed due to poor perfusion of the ipsilateral limb and persisting neuropathic ulcer. The patient's past medical history included diabetes mellitus type II, peripheral vascular disease requiring left femoral vascular intervention, and hypertension.

### 2.2. Examination

The patient was able to mobilize short distances with an antalgic gait. She was independent with activities of daily living but dependent on a wheelchair for outdoor ambulation. There was a significant leg length discrepancy with the left lower limb shortening by 4 cm. Soft tissues overlying the surgical site were intact. The patient's hip was irritable to movement. Peripheral pulses were present but diminished with sensory changes to the lower limb.

## 3. Surgery and Follow-Up

Intraoperatively, the femoral head was identified via posterior approach after extensive arthrolysis with a well-fixed femoral stem. The screw-type acetabular component was laboriously salvaged as it was surrounded by arthrofibrosis and heterotopic ossification. The acetabular defect was extensively debrided with multiple sampling of periprosthetic tissue for microbiology. These were negative for infection.

**Figure 2:**
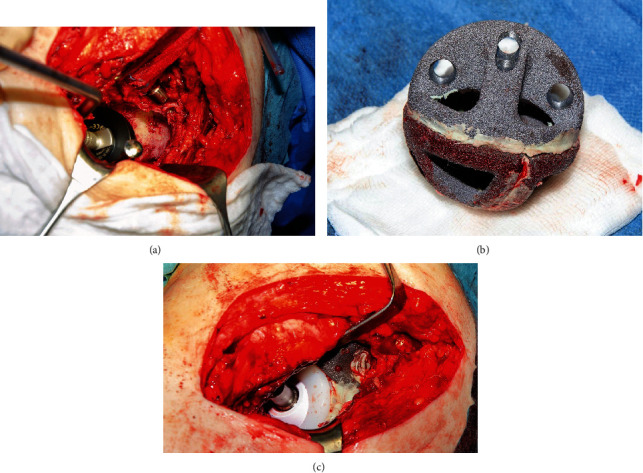
(a) Intraoperative photo of the acetabulum with the trial cup in situ in the original hip center. Large secondary spherical bone defect evident overlying the previous pseudoarthrosis. (b) Intraoperative photo using three tantalum augments and cementing them together in a “football configuration.” (c) Intraoperative photo showing the tantalum augments in situ filling the cranial acetabular defect. Underneath, we see the cemented Mark III cup with the ceramic head.

The remaining acetabulum consisted of a wafer-thin medial acetabular wall and a large supra-acetabular defect, consistent with a Paprosky Type IIIb defect [19] (Figure 2(a)). There were spherical acetabular defects over the true acetabulum and another spherical acetabular defect overlying the debrided pseudoarthrosis. Multiple configurations of the 54 mm tantalum augments (Zimmer Biomet, Warsaw, USA) were trialled to contain the acetabular defect. A novel method of acetabular reconstruction involves using three tantalum augments and cementing them together in a “football configuration” using regular bone cement (polymethylmethacrylate, PMMA) (Figure 2(b)). After keeping these three wedges in the correct position for twelve minutes until bone cement fixation was completed, the construct was then implanted into the acetabular defect and subsequently fixed with additional 6.5 mm cancellous bone screws.

**Figure 3:**
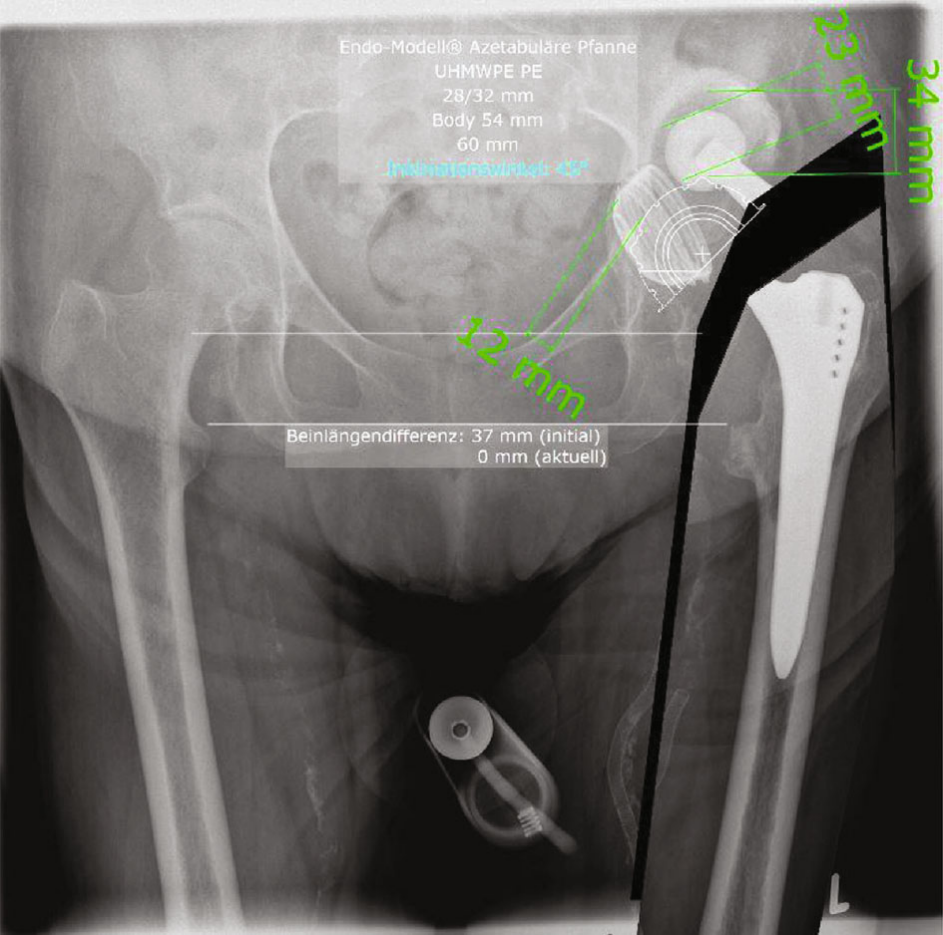
Preoperative planning using cemented Mark III cup.

**Figure 4:**
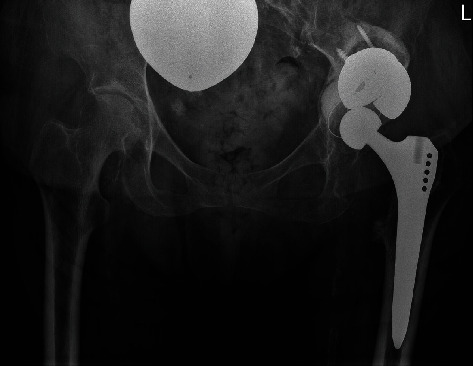
Postoperative pelvic overview with acetabular defect filling using three tantalum augments in a “football” configuration and reconstruction of the hip center of rotation.

Reconstruction of the acetabulum to achieve a physiological center of rotation was achieved using a cemented Mark III acetabular cup (Waldemar Link Company, Hamburg, Germany) according to preoperative planning (Figure 3). A compatible Delta ceramic head size 28 × 3, 0 mm (Biolox®, CeramTec, Plochingen, Germany) was placed on the original stem and achieved stable range of motion of the hip (Figures 2(c) and 4).

**Figure 5:**
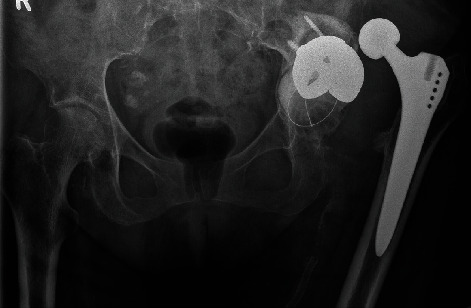
Follow-up X-ray demonstrating left hip dislocation at 4 weeks postop.

The patient was closely monitored within a multidiscipline in our institution, however sustained two dislocations during the postoperative period (Figure 5).

**Figure 6:**
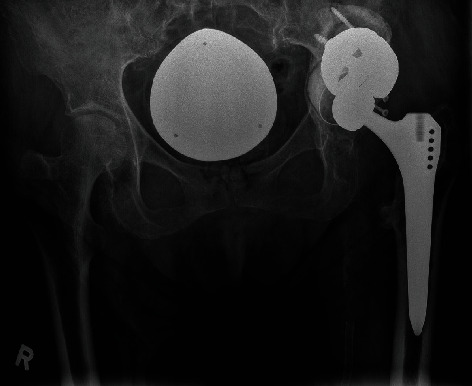
Last X-ray of the patient in clinic after the cemented cup revision. No further episodes of left hip dislocation.

Thus, the cemented cup was revised 4 weeks after the initial reconstruction with an elevated liner and installed an antidislocation ring (Figure 6) [12]. The patient did not sustain further dislocations and achieved independent mobility at the last follow-up.

## 4. Discussion

This case shows an individual and uncommon solution of acetabular bone defect reconstruction using multiple tantalum wedges which were fixed together via bone cement in a “three-quarter football” way. After a regular intrahospital stay, we postoperatively observed two hip joint dislocations which led to revision surgery.

Most common reasons for hip joint dislocation are either implant-related, like failed implant positioning, or soft tissue-related, like periarticular tension [20, 21]. In this case, the cemented implants were well fixed, so that we had to assume that the reason for joint dislocation was a tension problem. Looking at the patient's history with a hip joint dislocation time minimum of two years and a concomitant leg shortening of approximately 4 cm, joint tension was also no relevant problem here.

To gain the most effective dislocation safety while avoiding too severe surgery in this old and sick patient, we decided to not perform an implant exchange like using a dual-mobility cup but to perform a liner exchange and to install an antidislocation ring. Luckily, the patient did not experience any further dislocation afterwards.

Concerning the innovative achievement of this maneuver, the fixation of multiple tantalum wedges for ideal bone defect reconstruction indeed describes a novel technique. To the best of the authors' knowledge, such a procedure has not been described before. An advantage of this bone defect filling might be that the surgeon does not have to ream the whole acetabulum for installing a dual-mobility cup or a jumbo cup, for example. This individual defect fitting might save patients' bone stock while still providing revision options in case of possible implant loosening during long-term follow-up.

Using multiple wedges for bone defect reconstruction has been described in a different way earlier with good preliminary results [22]. Though tantalum implants seem to have a quite high overall long-term survival [13, 23], the weakness of these cases is a missing long-term follow-up.

## 5. Conclusions

This IS a case of complex acetabular defect addressed with a novel method of acetabular reconstruction using multiple tantalum augments. The described method allows modular reconstruction of large spherical defects overlying the true and false acetabulum by creating a tantalum “football” augment provisionally hold together with bone cement. The patient achieved good function despite the complexity associated with a chronically dislocated total hip replacement and formation of pseudoarthrosis.
